# Air travel demand forecasting based on big data: A struggle against public anxiety

**DOI:** 10.3389/fpsyg.2022.1017875

**Published:** 2022-12-05

**Authors:** Xiaozhen Liang, Chenxi Hong, Wenkun Zhou, Mingge Yang

**Affiliations:** School of Management, Shanghai University, Shanghai, China

**Keywords:** public anxiety, big data mining, air travel demand, convolutional LSTM, artificial intelligence algorithm

## Abstract

It is of great significance to accurately grasp the demand for air travel to promote the revival of long-distance travel and alleviate public anxiety. The main purpose of this study is to build a high-precision air travel demand forecasting framework by introducing effective Internet data. In the age of big data, passengers before traveling often look for reference groups in search engines and make travel decisions under their informational influence. The big data generated based on these behaviors can reflect the overall passenger psychology and travel demand. Therefore, based on big data mining technology, this study designed a strict dual data preprocessing method and an ensemble forecasting framework, introduced search engine data into the air travel demand forecasting process, and conducted empirical research based on the dataset composed of air travel volume of Shanghai Pudong International Airport. The results show that effective search engine data is helpful to air travel demand forecasting. This research provides a theoretical basis for the application of big data mining technology and data spatial information in air travel demand forecasting and tourism management, and provides a new idea for alleviating public anxiety.

## Introduction

Today’s world is in an unprecedented upheaval. The whole world is suffering from various psychosocial problems under the catalysis of various unstable factors. For example, from the perspective of interpersonal relationship, the occurrence of the aggregation epidemic has led to the interpersonal relationship being fragility, alienation and lack of trust (Mei et al., 2022); and from the perspective of individual pressure, the terrible economic environment and severe employment pressure have forced individuals to lower their expectations of life, whose pessimism have pervaded the whole society (Lu and Lin, 2021). Many researchers have conducted process analysis and means discussion on the adjustment of social psychological state from different perspectives, such as tourism (Chen et al., 2020), community positive psychological intervention (Montiel et al., 2021), and localized constructive news (Ouyang et al., 2021). Among them, tourism as a “spiritual product,” can not only meet complexity requirements of people (i.e., the pursuit of novelty, change and unpredictability) to strongly stimulates their dopamine, but also drive consumption and promote economic recovery (Hu et al., 2022). In addition, taking into account the freshness of scenic spots outside the province for people, long-distance travel can often generate more powerful energy (Hannah and Lisa, 2020). As an important way of long-distance travel, aviation plays a pivotal role. It is of great significance to accurately predict the future air travel demand, which contributes to not only carry out infrastructure construction, emergency plan adjustment and resource scheduling for the government, but also reasonable operation strategies formulation for tourism companies to improve the quality of tourism services, passenger satisfaction and security (Fildes et al., 2011). Therefore, improving the accuracy of air travel demand forecasting can be regarded as a new idea to promote the recovery of long-distance travel and alleviate public anxiety, which has great research value.

**Figure 1:**
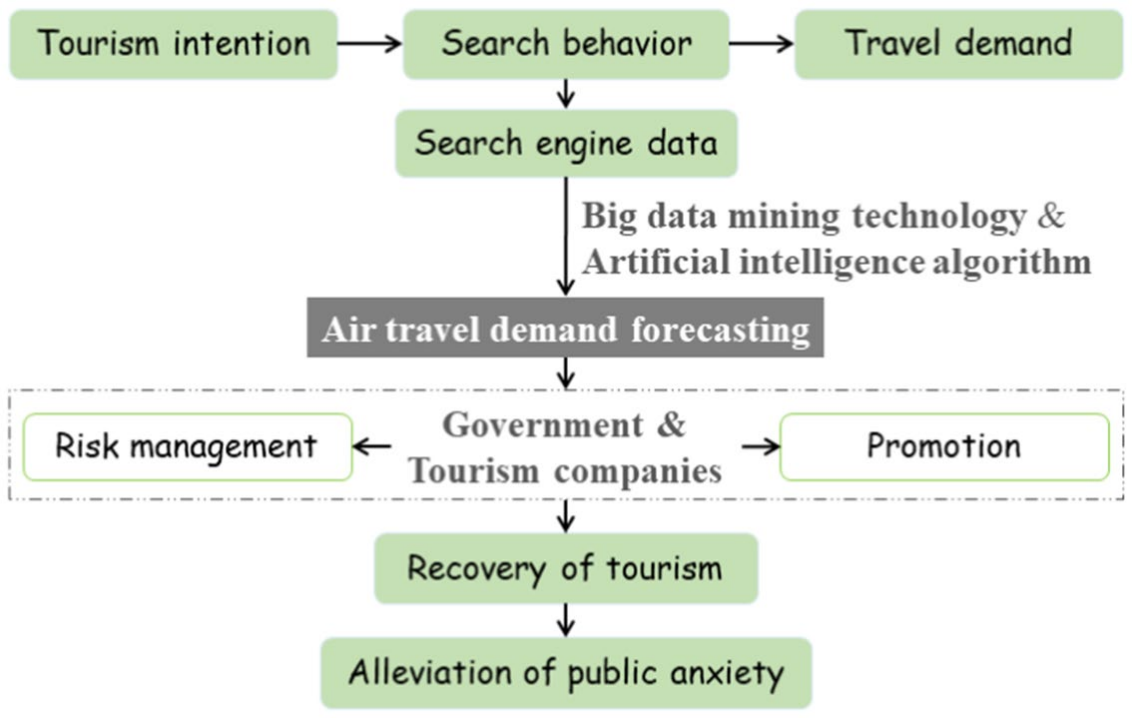
The underlying logic of the research.

Most existing studies on air travel demand forecasting have always been dependent on traditional historical statistical data (Jin et al., 2020), which is released with low frequency and only reflects relatively objective information on a macro level. In the era of Internet big data, as a matter of fact, data from Internet search queries provide a new source of predictive variables. When tourists generate tourism intentions about a destination, they need to have a deep understanding of the object due to their knowledge seeking psychology. And the more frequently tourists search for information, the stronger the correlation between their tourism behavior and tourism intention (Tavitiyaman et al., 2021). Therefore, the overall tourism intention reflected by the search engine data, a kind of big data, is also highly correlated with the social travel demand. A large number of existing studies indicate that the predictive accuracy will be generally improved when search engine data of query keywords are used as explanatory variables in forecasting models (Liu et al., 2021; Aaronson et al., 2022). However, most of the previous literature have ignored the damage to the prediction accuracy caused by much noise information contained in big data. In this paper, we aim to explore how to make use of this big data in turn to achieve better air travel demand forecasting performance when the Internet is gradually dominating people’s lives. Meanwhile, the novel idea is provided for the recovery of tourism and the alleviation of public anxiety in the turbulent era. The underlying logic of this study is shown in Figure 1.

In addition, the nonlinear and dynamic data characteristics of air travel demand and explanatory factors increase the problem complexity. In recent years, the common forecasting methods are mainly divided into traditional econometric models and artificial intelligence models. Traditional econometric models, such as the autoregressive integrated moving average (ARIMA) model (Jimenez et al., 2022) and the autoregressive distribution lag (ARDL) model (Madziwa et al., 2022), can capture the relationships between historical and future values to obtain better prediction results. Because of their simple principle and easy implementation, they are widely used to forecast time series to obtain high prediction accuracy for stationary time series. On the contrary, advanced artificial intelligence algorithms show strong nonlinear mapping abilities. For example, Xiong et al. (2021) designed a forecasting framework with the support vector regression (SVR), whose feasibility and effectiveness have been proved in experiments. Chen and Huang (2021) used convolutional neural network (CNN) and long short-term memory network (LSTM) respectively to implement stock price prediction based on eight input features, which fully proved the superiority of artificial intelligence models in forecasting complex time series. However, the above models are mostly used to analyze and extract the temporal relationship of data rather than the spatial relationship for air travel demand forecasting. In fact, the spatial effect of travel flows can occur and have an impact on adjacent destinations (Chhetri et al., 2013). Therefore, the spatial effect has great potential to assist the forecasting for air travel demand. The existing literature has proved the spatial correlation between tourism flows in adjacent areas (Yang and Zhang, 2019; Jiao et al., 2020; Ouchen and Montargot, 2021). However, there are few researches to make full use of the spatial correlation of series to assist air travel demand forecasting. Expanding to other fields, Zhang et al. (2021) embedded CNN model and LSTM model into traffic generative adversarial nets to deeply capture temporal and spatial correlation between road connections for road network level prediction. Fu et al. (2022) proposed a wind power prediction method considering the spatial distribution characteristics of field groups based on LSTM model and time convolution neural network (TCN). Shi et al. (2015) proposed a new method namely convolutional LSTM (ConvLSTM), which organically combines the convolutional operator with LSTM to complete the learning of spatial–temporal information in essence. Above studies have confirmed that the introduction of spatial dependence of input data is conducive to improving the performance of prediction models, and the combination of LSTM model and CNN model is a feasible choice for processing spatial–temporal data. Compared with other combination methods, ConvLSTM model can achieve deeper learning of spatial data relevance through simple model structure. Therefore, this research used ConvLSTM model to simultaneously analyze the spatial and temporal dependence of the search engine data reflecting the spatial effect of tourism flow and the historical data of air travel demand, so as to capture more effective information for assisting prediction.

Moreover, traditional econometric models and artificial intelligence models have their own advantages and disadvantages so that cannot give full play to all the advantages when processing the input data with complex components. At present, the ensemble prediction model which combines decomposition method and prediction model is generally regarded as an effective method (Li et al., 2022). More precisely, the decomposition method contributes to exploration about information of different time scales of the series pointedly to generate better prediction. In this paper, a novel decomposition algorithm named time varying filtering-based empirical mode decomposition (TVF-EMD) was applied to disassemble the information of different scales of the original sequence, which can use time-varying filtering to alleviate the mode mixing problem, and produce the decomposition results that have strong stability (Liu et al., 2022). To sum up, based on the ensemble framework, this paper attempts to use ConvLSTM model as the main prediction model, and combine the effective search engine data that can make full use of the search psychology of passengers and the spatial effect of tourism flow to achieve high-precision air travel demand prediction. Through this design of forecasting framework, the prediction accuracy of air travel demand has the opportunity to increase significantly.

The main contributions of this paper include: (1) From the perspective of improving the prediction accuracy of air travel demand, it has developed a new direction for the recovery of tourism and the alleviation of public anxiety by providing decision support for putting forward reasonable policies and measures; (2) Based on the designed dual data preprocessing method, effective search engine data is introduced, which can help to enhance the accuracy of air travel demand forecasting model; (3) The concept of spatial effect of tourism flow is first introduced into the field of air travel demand forecasting, and the ConvLSTM model is introduced to fully extract the spatial–temporal characteristics of search engine data and historical data; (4) An ensemble forecasting framework is established by combining the econometric model with artificial intelligence model for air travel demand, which can fully avoid the impact of market environment and give play to the advantages of different models to improve the prediction performance.

The rest work is designed as follows. Section “Methodology” presents the approaches used in the ensemble forecasting framework. Section “Overall forecasting framework” provides design ideas and detailed information about the overall framework of the proposed model. Section “Experimental process” describes the process of data preprocessing and prediction as necessary. In Section “Discussion,” the experimental results are introduced, and the deep insights about how to make practical contributions to social well-being interventions based on the proposed model are provided. Section “Conclusion” summarizes the conclusions of this study.

## Methodology

### Proposed dual data preprocessing method

#### First-stage data preprocessing

The herd mentality and curiosity seeking mentality of most Internet users will lead to the rapid evolution of a novel event into a popular search keyword on the Internet, even if this event has nothing to do with their travel intention. As a result, the collected search engine data contains some outliers and noise, which need to be eliminated in advance. Scrupulously, two algorithms are introduced to remove the outliers and noises of initial SED:

1. K-means clustering algorithm. It is a classic partition clustering method proposed by Macqueen (1967). For a given dataset D containing n objects in Euclidean space, K-means algorithm will divide the data objects in D into k clusters $C_{1},C_{2},\ldots ,C_{k}\text{.}$ The goal of partition is to make the objects within a cluster have high similarity while the objects between clusters have low similarity. In this paper, the data excluded from large clustering by K-means algorithm are considered as outliers.

The basic flow of the algorithm is described as follows:

Step 1: Set k as the number of clustering centers in advance, and input the dataset $D=\left\{x_{i}|x_{i}\in R^{m},i=1,2,\ldots ,n\right\}$ containing n data samples.

Step 2: *k* sample points are randomly selected as the initial clustering centers, denoted as $y_{1},y_{2},\ldots ,y_{k}\text{.}$

Step 3: Calculate the Euclidean distance between each sample point $x_{i}$ and each clustering center $y_{j}$, and the calculation formula is as follows:


(1) $$\begin{array}{l} d\left(x_{i},y_{j}\right)=\\ \;\sqrt{{\left(x_{i1}-y_{j1}\right)}^{2}+{\left(x_{i2}-y_{j2}\right)}^{2}+\ldots +{\left(x_{im}-y_{jm}\right)}^{2}} \end{array}$$

According to the distance, each point $x_{i}$ is placed into the corresponding cluster $C_{j}$ to minimize the distance between $x_{i}$ and $y_{j}$.

Step 4: Calculate the criterion function E for the sum of error squares after clustering, and the formula is as follows:


(2) $$E=\overset{k}{\underset{j=1}{\sum }}\underset{x_{i}\in C_{j}}{\sum }d\left(x_{i},y_{j}\right)$$

Step 5: For each cluster $C_{j}$, recalculate its new cluster center, the formula is as follows:


(3) $$y_{j}=\frac{1}{\left|C_{j}\right|}\underset{x_{i}\in C_{j}}{\sum }x_{i}$$

where $|C_{j}|,\left(j=1,2,\ldots ,k\right)$ is the number of points in the cluster $C_{j}$ that completed the allocation.

Step 6: Based on the new clustering centers, recalculate the criterion function $E^{\prime }$. If $\left|E-E^{\prime }\right|$ is greater than the specified threshold, repeat steps 3–5. Otherwise, the algorithm ends to output the clustering result.

(2) TVF-EMD decomposition algorithm. By definition, a single-component signal with a specific physical interpretation is called an intrinsic mode function (IMF). The traditional EMD decomposition algorithm has an inherent defect namely mode mixing problem, that is, a certain IMF generated by decomposition has different scales, or different IMFs appear on the same scale (Zheng et al., 2017). In this context, TVF-EMD proposed by Li et al. (2017) attempts to define the IMF from another perspective and adjust the cut-off frequency to overcome the mode mixing problem. Specifically, TVF-EMD algorithm will successively extract qualified sub-sequences, namely IMF components, through a series of calculation formulas. Due to space limitations, this paper only briefly describes the operation process as follows.

Step 1: Calculate the instantaneous amplitude $A\left(t\right)$ and instantaneous phase $\varphi \left(t\right)$ of the actual signal $x\left(t\right)$:


(4) $$A\left(t\right)=\sqrt{x^{2}\left(t\right)+{\hat{x}}^{2}\left(t\right)}$$

(5) $$\varphi \left(t\right)=\arctan\left[\hat{x}\left(t\right)/x\left(t\right)\right]$$

where $\arctan\left[\;\right]$ is the inverse tangent function, and $\hat{x}\left(t\right)$ is the Hilbert transform of $x\left(t\right)$.

Step 2: Calculate all local maximum values *A*({*t_max_*}) and minimum values *A*({*t_min_*}) of *A*(*t*), respectively.

Step 3: The upper envelope $\beta_{1}\left(t\right)$ and the lower envelope $\beta_{2}\left(t\right)$ are estimated respectively, and then the amplitudes $a_{1}\left(t\right)$ and $a_{2}\left(t\right)$ are calculated based on the following formulas:


(6) $$\left\{ \begin{array}{c} a_{1}\left(t\right)=\left[\beta_{1}\left(t\right)+\beta_{2}\left(t\right)\right]/2\\ a_{2}\left(t\right)=\left[\beta_{1}\left(t\right)-\beta_{2}\left(t\right)\right]/2 \end{array}\right.$$

Step 4: Based on a series of points $\varphi^{\prime }\left(\left\{t_{\text{min}}\right\}\right)A^{2}\left(\left\{t_{\text{min}}\right\}\right)$ and $\varphi^{\prime }\left(\left\{t_{\text{max}}\right\}\right)A^{2}\left(\left\{t_{\text{max}}\right\}\right)$ for interpolation respectively, accordingly estimate $\eta_{1}\left(t\right)$ and $\eta_{2}\left(t\right)$, so as to calculate the instantaneous frequencies $\varphi_{1}^{\prime }\left(t\right)$ and $\varphi_{2}^{\prime }\left(t\right)$. The mathematical expressions are as follows:


(7) $$\left\{ \begin{array}{c} \begin{array}{l} \varphi_{1}^{\prime }\left(t\right)=\eta_{1}\left(t\right)/\left(2a_{1}^{2}\left(t\right)-2a_{1}\left(t\right)a_{2}\left(t\right)\right)+\\ \eta_{2}\left(t\right)/\left(2a_{1}^{2}\left(t\right)+2a_{1}\left(t\right)a_{2}\left(t\right)\right) \end{array}\\ \begin{array}{l} \varphi_{2}^{\prime }\left(t\right)=\eta_{1}\left(t\right)/\left(2a_{2}^{2}\left(t\right)-2a_{1}\left(t\right)a_{2}\left(t\right)\right)+\\ \eta_{2}\left(t\right)/\left(2a_{2}^{2}\left(t\right)+2a_{1}\left(t\right)a_{2}\left(t\right)\right) \end{array} \end{array}\right.$$

Step 5: Calculate the local cut-off frequency $\varphi_{\mathrm{bis}}^{\prime }\left(t\right)$:


(8) $$\varphi_{\mathrm{bis}}^{\prime }\left(t\right)=\left[\varphi_{1}^{\prime }\left(t\right)+\varphi_{2}^{\prime }\left(t\right)\right]/2=\left(\eta_{2}\left(t\right)-\eta_{1}\left(t\right)\right)/4a_{1}\left(t\right)a_{2}\left(t\right)$$

Step 6: Rearrange $\varphi_{\mathrm{bis}}^{\prime }\left(t\right)$ based on local cut-off frequency realignment.

Step 7: Calculate signal $h\left(t\right)$ accordingly:


(9) $$h\left(t\right)=\cos\left[\int \varphi_{\mathrm{bis}}^{\prime }\left(t\right)dt\right]$$

$x\left(t\right)$ is filtered by B-spline approximation, and the approximate result is $m\left(t\right)$.

Step 8: Update$x\left(t\right)$ according to the specified rules. That is, if $\theta \left(t\right)\leq \xi$, $x\left(t\right)$ is considered as an IMF component and the calculation is terminated. Otherwise, set $x\left(t\right)=x\left(t\right)-m\left(t\right)$, and repeat Step 1–Step 7. The calculation formula of the stopping criterion $\theta \left(t\right)$ is as follows:


(10) $$\theta \left(t\right)=B_{\mathrm{Loughlin}}\left(t\right)/\varphi_{avg}\left(t\right)$$

where $\varphi_{avg}\left(t\right)$ is the weighted average of instantaneous frequencies, and $B_{\mathrm{Loughlin}}\left(t\right)$ is *Loughlin* instantaneous bandwidth. Until the IMF is no longer generated, the remainder is denoted as a residue (R). Then, the IMF with the highest frequency in TVF-EMD decomposition results is considered as noise information (Li et al., 2017).

#### Second-stage data preprocessing

In order to develop search engine data that have real predictive ability for air travel demand as explanatory variables, it is necessary to adopt appropriate filtering methods to process the search engine data. Specifically, the augmented Dickey-Fuller (ADF) test, Engle-Granger (E-G) cointegration test, Granger causality test are used to select the variables with high forecasting ability for air travel demand (Huang et al., 2017). After the above statistical tests, the selected explanatory variables will have the same stability as the predicted variables, and show a strong long-term cointegration relationship and causality with the predicted variables. Moreover, Pearson correlation coefficient analysis (Lai et al., 2022) is introduced to select the objects with relatively high correlation with the predicted variables from the variables that pass the statistical test. It is worth mentioning that Pearson correlation coefficient is the most commonly used statistical indicator to reflect the degree of correlation between variables.

### Convolutional long short-term memory network

#### Long short-term memory network

**Figure 2:**
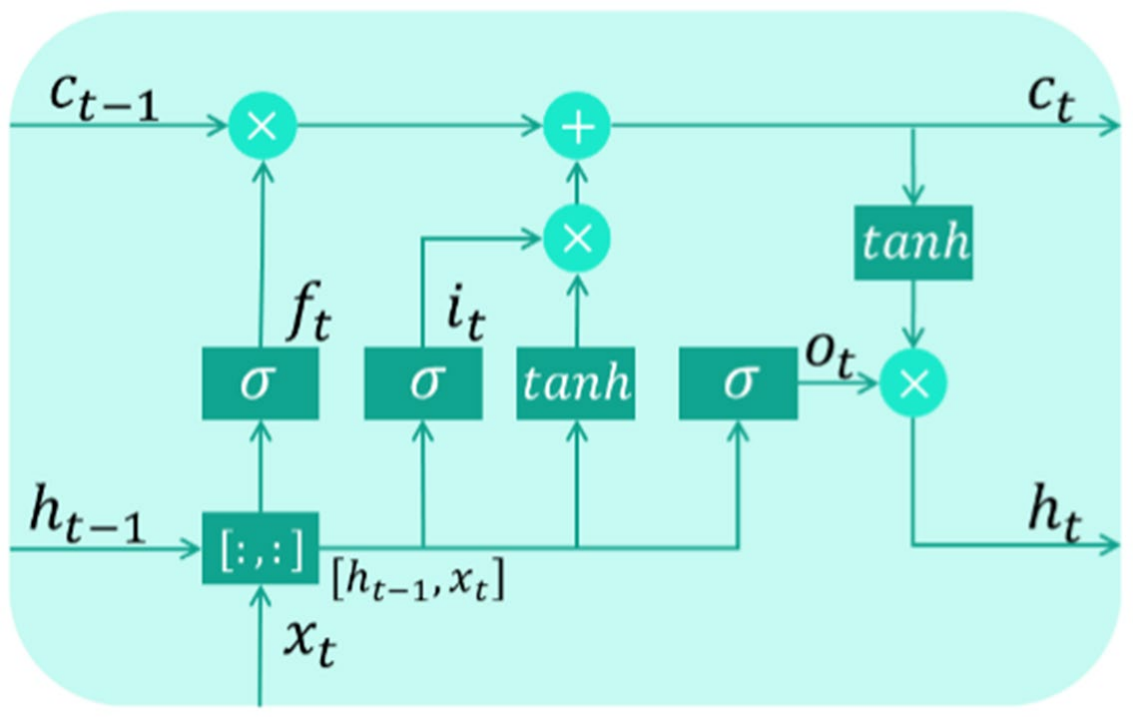
Structure of a LSTM memory cell.

The structure of the LSTM (shown in Figure 2) determines that it is capable of solving the problem namely long-term dependence to achieve satisfactory prediction results.

Figure 2 shows that the input gate $i_{t}$, forget gate $f_{t}$, and output gate $o_{t}$ constitute the main structure of LSTM. After being activated, $i_{t}$ can selectively store new input information, $f_{t}$ can omit unnecessary information, and $o_{t}$ can control the part of output. Finally, the current output $h_{t}$ is updated. The above process can be expressed as follows:


(11) $$f_{t}=\sigma \left(W_{f}\circ \left[h_{t-1},\;x_{t}\right]+b_{f}\right)$$

(12) $$i_{t}=\sigma \left(W_{i}\circ \left[h_{t-1},\;x_{t}\right]+b_{i}\right)$$

(13) $$o_{t}=\sigma \left(W_{o}\circ \left[h_{t-1},\;x_{t}\right]+b_{o}\right)$$

(14) $$c_{t}=f_{t}\circ c_{t-1}+i_{t}\circ \tanh\left(W_{c}\circ \left[h_{t-1},\;x_{t}\right]+b_{c}\right)$$

(15) $$h_{t}=o_{t}\circ \tanh\left(c_{t}\right)$$

where $W_{f}$, $W_{i}$, $W_{o}$ and $W_{c}$ are weight vectors, $b_{f}$, $b_{i}$, $b_{o}$, and $b_{c}$ are bias vectors, $x_{t}$ is the current input, $h_{t-1}$ represents the output of the previous moment, $c_{t}$ and $c_{t-1}$ are memory units of the current moment and the previous moment, respectively. In addition, $\sigma \left(•\right)$ and $\tanh\left(•\right)$ represent sigmoid function and hyperbolic tangent activation function respectively, and ∘ stands for the Hadamard operator.

#### Convolutional long short-term memory network

The ConvLSTM model is an improved version of the LSTM model. The key point of its improvement is to replace the Hadamard operators with the convolutional operators for both the input-to-state transformations and the state-to-state transformations, which makes the network enable to fully learn the hidden correlations in spatial–temporal data through limited historical data. The functions used in LSTM are modified for ConvLSTM, as shown below:


(16) $$f_{t}=\sigma \left(W_{f}*\left[h_{t-1},\;x_{t}\right]+b_{f}\right)$$

(17) $$i_{t}=\sigma \left(W_{i}*\left[h_{t-1},\;x_{t}\right]+b_{i}\right)$$

(18) $$o_{t}=\sigma \left(W_{o}*\left[h_{t-1},\;x_{t}\right]+b_{o}\right)$$

(19) $$c_{t}=f_{t}\circ c_{t-1}+i_{t}\circ \tanh\left(W_{c}*\left[h_{t-1},\;x_{t}\right]+b_{c}\right)$$

(20) $$h_{t}=o_{t}\circ \tanh\left(c_{t}\right)$$

where ∗ represents the convolutional operator.

## Overall forecasting framework

Tourism can not only directly stimulate people’s positive emotions, but also indirectly relieve public pressure by stimulating local economic growth (Liu et al., 2021). It is necessary to master the future air travel demand for formulating reasonable policies and measures to promote long-distance tourism. Therefore, the main purpose of this paper is to build a high-precision air travel demand forecasting framework.

In the Internet era, online search engine data can represent people’s behavior and generate early indicators of future demand to a certain extent (Huang and Hao, 2021). With the rapid popularization of the Internet, search engine has become one of the primary means of modern information searching, such as Baidu and Google. Baidu and Google generate search engine data of each keyword (i.e., Baidu Index and Google Trend) based on the search volume of hundreds of millions of users according to specific formulas, this is, the weighted sum of the search times of each keyword in their search engines (Yao et al., 2021). Both the Baidu Index (Liang et al., 2022) and Google Trend (Sun et al., 2022) can reflect the potential demand and the current focus. Considering that this paper studies the forecast of air travel demand in China, Baidu Index is a better choice.

Tourism preference is considered as an important psychological tendency (Yu and Lu, 2008). To be specific, people generally need to investigate and understand the information of tourist products, such as attractions, delicacies and overall situation, so as to determine the emotional tendency of tourist products and generate the intention of yearning or repelling. In the big data era, passengers are used to querying information in search engines before making travel plans. The relevant big data records the attention of passengers to the destination and their desire to travel to a certain extent. Therefore, appropriate search engine data can reflect the air travel demand of destinations in advance. Furthermore, considering that the travel flows of adjacent areas will have a spatial effect on it of the destination, this paper tries to obtain the required search engine data by using “name,” “name + attraction” and “name + delicacies” of the destination where the airport is located and its adjacent destinations as seed keywords.

With the prevalence of new media, social self-media applications gradually carve up the market share of search engines in information query, which may lead to the change of air travel demand being reflected incorrectly by the change of the later trend of search engine data. In order to introduce available fluctuation information and eliminate unstable trend information of search engine data, decomposition-ensemble strategy can be adopted to distinguish linear trend term and nonlinear fluctuation term of variables, and appropriate prediction models can be used for analysis separately.

**Figure 3:**
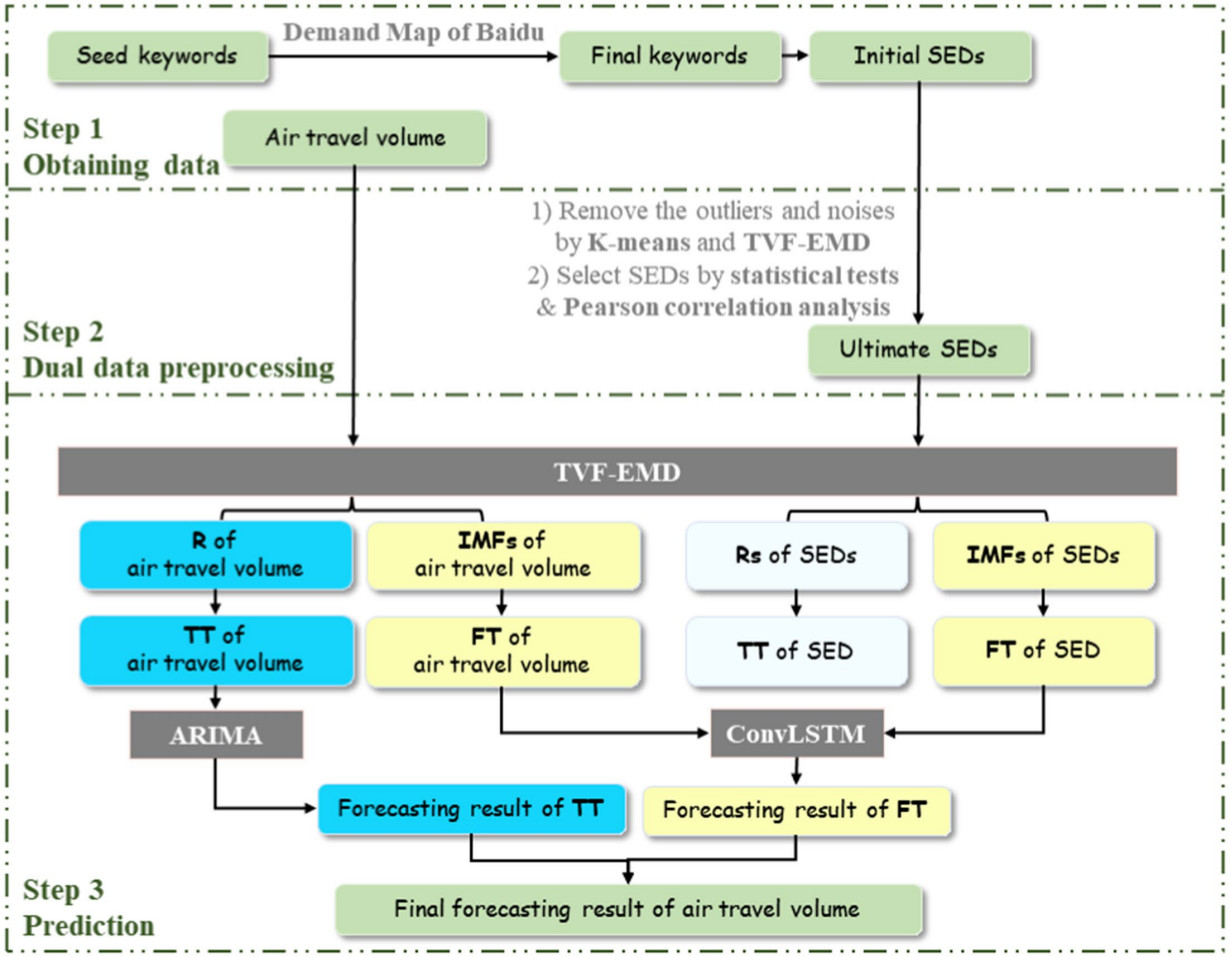
The structure of SED-DDP-TVFEMD-ConvLSTM ensemble forecasting framework.

Based on above analysis, the proposed ensemble forecasting framework for air travel demand combining search engine data (SED), dual data preprocessing (DDP), TVF-EMD and ConvLSTM, namely SED-DDP-TVFEMD-ConvLSTM, is shown in Figure 3.

The major procedures are demonstrated as follows:

Step 1: All seed keywords are selected and searched on Baidu Index. The search volumes of these keywords and their associated keywords are found out through the Demand Map function of Baidu Index. After removing repetitive keywords and the keywords with less search, the final keywords are determined.

Step 2: The proposed DDP method is used to clean initial SEDs, including removing outliers and noises by K-means and TVF-EMD algorithms, and selecting effective SEDs by statistical tests and Pearson analysis.

Step 3: First, the TVF-EMD algorithm is applied to decompose each variable into a set of sub-sequences (i.e., IMFs and R). Then, for each variable, the R component with the lowest frequency is taken as the trend term (TT), and all IMFs are reconstructed into the fluctuation term (FT). In addition, considering that collinearity among multiple SEDs may lead to over-fitting problems in the forecasting framework, the TTs of several SEDs are further integrated into a TT component, as well as the FTs of several SEDs into a FT component, to replace the scattered SEDs to express the travel flows in the surrounding areas antecedently. Based on this, two components of air travel volume and SED can be obtained, respectively. Next, ARIMA model is employed to model and forecast the TT component of air travel volume, and ConvLSTM model is utilized to forecast the FT component of air travel volume based on the FTs of air travel volume and SED. The final forecasting result of air travel volume is obtained by simply adding the forecasting results of the FT and TT components.

## Experimental process

### Data preparation

#### Air travel volume data

**Figure 4:**
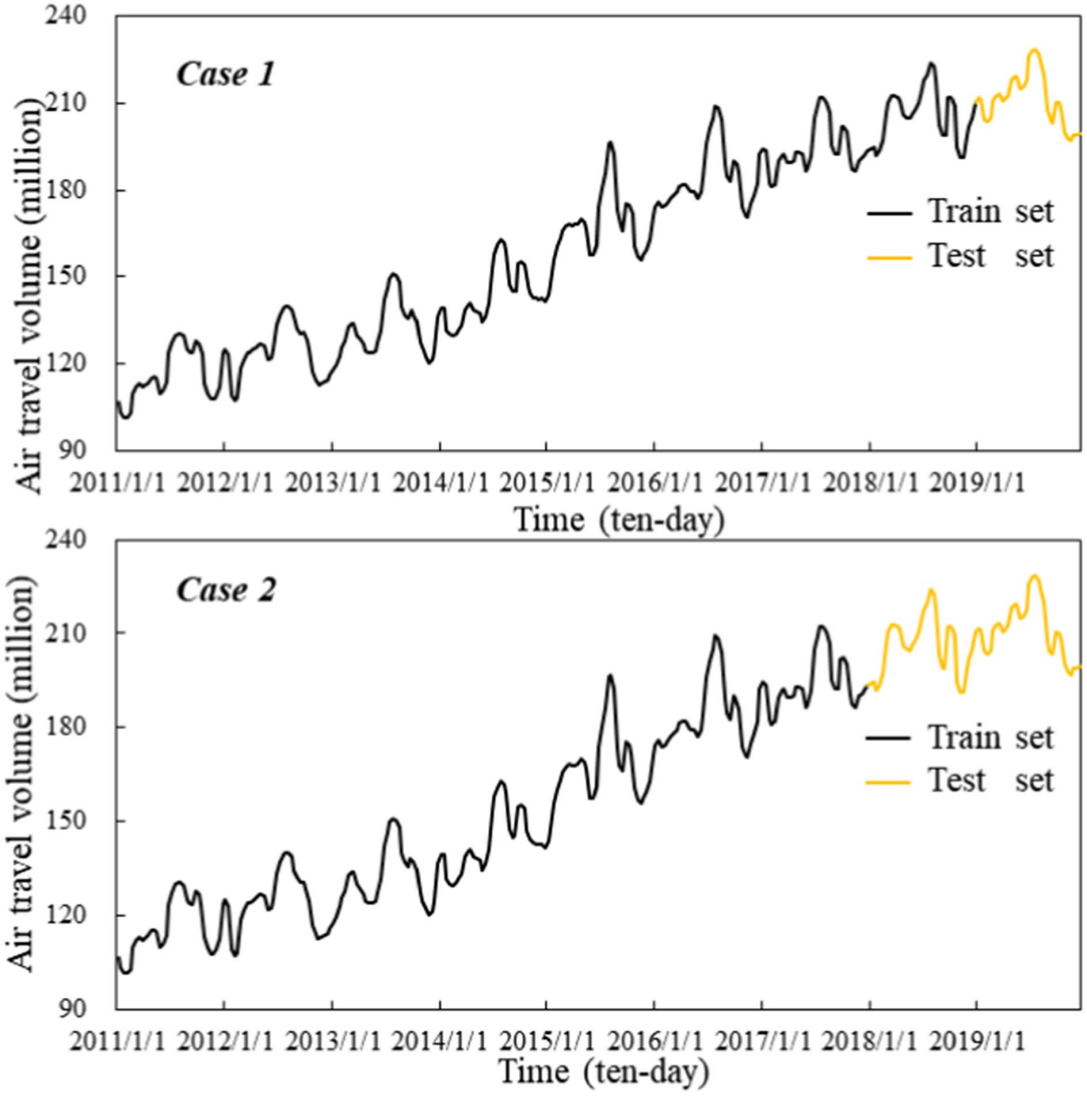
Air travel volume of Shanghai Pudong International Airport in two cases from January 2011 to December 2019.

In this study, the air travel volume data of Shanghai Pudong International Airport (ZSPD) was used as the experimental object to verify the positive effect of spatial–temporal search engine data and the validity of the proposed ensemble framework for air travel demand forecasting. Due to the epidemic situation, domestic airports were closed or semi closed for a long time from 2020 to the later stage, and the government had not released effective statistical data. Therefore, the data used in this paper only covers the period from January 2011 to December 2019. The required air travel volume data came from the Wind database^1^, and was low-frequency monthly data containing 108 observations. In order to further enhance the performance of ConvLSTM model, the monthly data was converted into 10-day data containing 324 observations by interpolation method (Xie et al., 2021). Additionally, this dataset was divided into train set and test set based on 8:1 (Case 1) and 7:2 (Case 2) ratios, respectively, to obtain two available datasets. The corresponding visualization representation is illustrated in Figure 4.

#### Spatial–temporal search engine data

##### Keywords confirmation

In order to collect SED that can represent the travel flows of cities around Shanghai, “names of 26 urban agglomerations in the Yangtze River Delta region,” “names + attraction” and “names + delicacies” were taken as seed keywords and entered into the search box of Baidu Index^2^ in turn. Then, the highly relevant sub-keywords were screened out by the Demand Map function continuously to expand the key thesaurus. Meanwhile, the irrelevant keywords were removed by virtue of experience. Finally, total 90 keywords were determined, and the corresponding SED data from January 2011 to December 2019 were obtained according to these keywords, which were further converted into 10-day data.

##### First-stage data preprocessing

To optimize SED quality, K-means clustering algorithm was first adopted to identify and delete outliers of SED groups in different cities. According to the Elbow curve, the optimal number of clustering centers for each group was determined, and the outlier ratio was set to 0.05. Then, the multiple imputation method and reliability analysis were combined to generate a complete alternative dataset.

**Figure 5:**
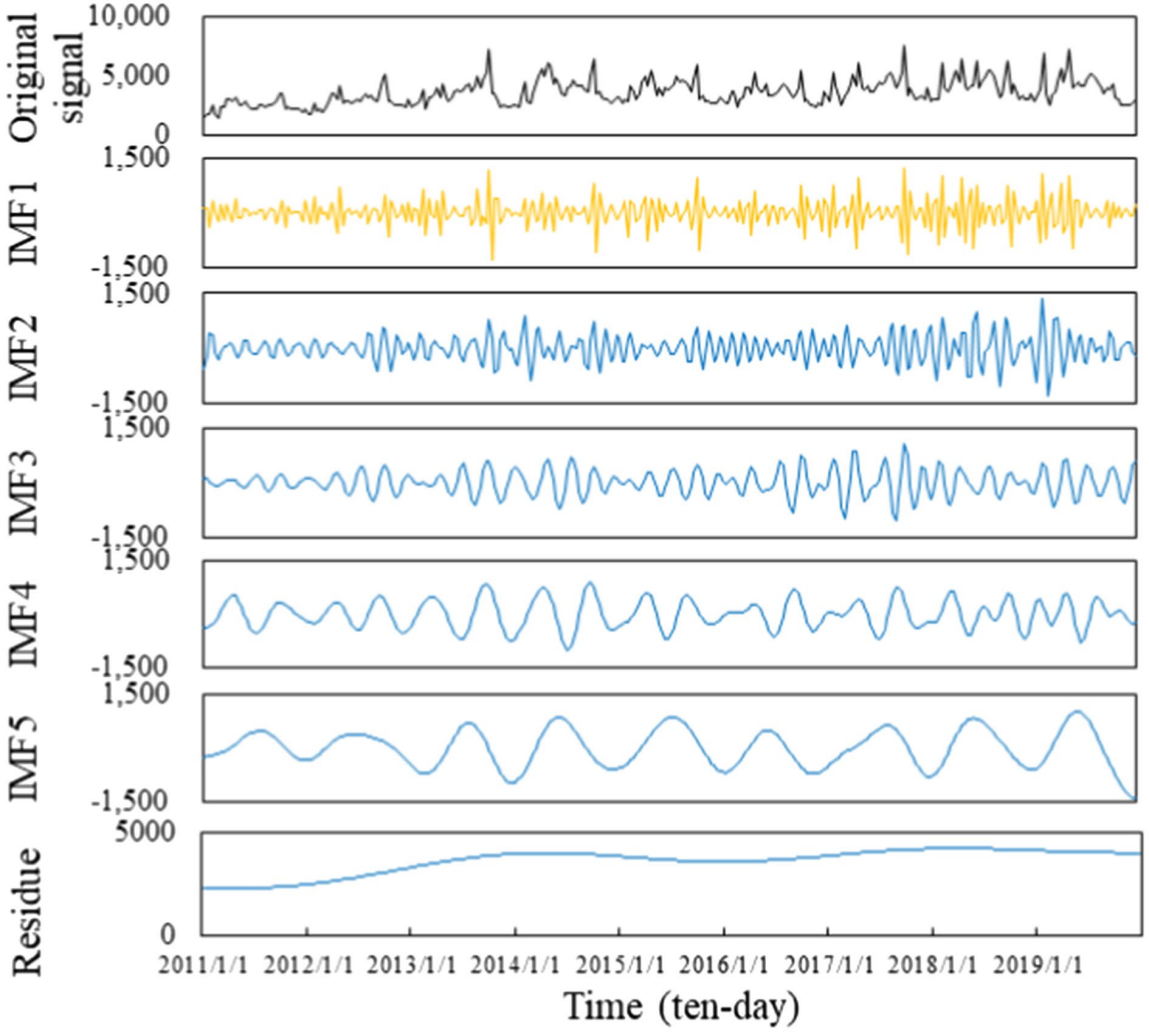
Decomposition results of “Ningbo Attraction” SED after TVF-EMD algorithm.

In addition, the TVF-EMD algorithm was used to decompose each SED sequence. Taking “Ningbo Attraction” SED as an example, the decomposition results are shown in Figure 5. It can be found that the high-frequency component IMF1 of the SED fluctuates violently and has a short period, which can be regarded as a random factor affecting the sequence. In other words, IMF1 contains more information unrelated to the air travel demand and no longer has the ability to predict the air travel demand. Hence, this component was directly rounded off. Furthermore, the remaining IMFs and residue were reconstructed into a new sequence, which contains the original available information.

##### Second-stage data preprocessing

In this section, standard statistical tests and correlation analysis were employed to select SED variables with predictive ability for air travel demand. Specifically, the ADF test was performed on the sequences firstly, and the SEDs whose stationarity was consistent with the predicted variable were retained. Subsequently, the E-G cointegration test was used to further test whether there was a long-term equilibrium relationship between SEDs and the predicted variable, and Granger causality test was used to observe whether SEDs could explain the future variation trend of the predicted sequence. Finally, the Pearson correlation coefficient was used to obtain the SEDs that contribute greatly to the forecast of airport travel demand. We set the keyword correlation coefficient threshold to 0.4, and then reserved the keywords whose correlation coefficients are higher than the threshold.

**Figure 6:**
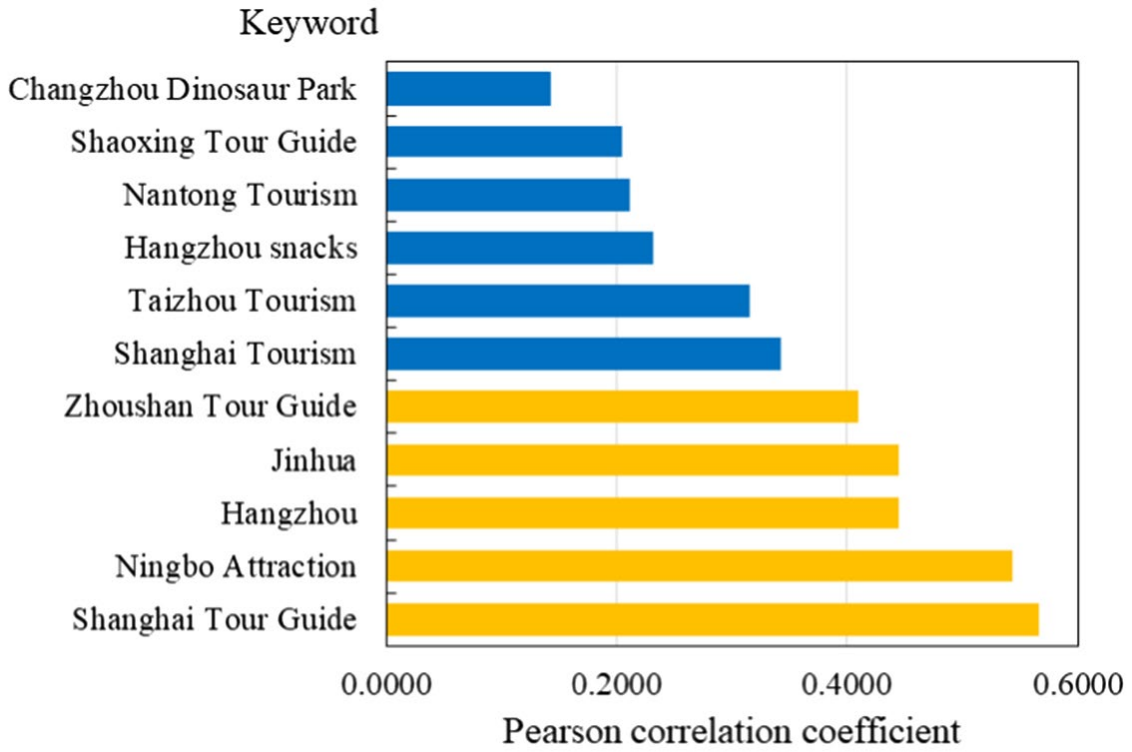
The Pearson correlation coefficients between partial SEDs and the predicted variable.

After dual data preprocessing, five SEDs were retained, that is, “Shanghai Tour Guide,” “Hangzhou,” “Ningbo Attraction,” “Jinhua” and “Zhoushan Tour Guide.” These SEDs all passed the tests at a significant level of 0.01, and the Pearson correlation coefficients between them and the predicted variable are shown in Figure 6.

### Prediction

Considering that different variables contain different time scales, the corresponding residue component for each variable decomposed by TVF-EMD algorithm was taken as the trend term (TT), and the remaining components were integrated as the fluctuation term (FT). To avoid the multicollinearity problem, the TT and FT components of the five SEDs were reconstructed, respectively, to obtain the final TT component and FT component of SED. Based on the introduction of SED as the explanatory variable, the FT component of air travel volume was predicted by ConvLSTM model, and the TT component of air travel volume was directly predicted by ARIMA. Finally, the predicted values of the FT and TT components were added to generate the final forecasting result of air travel volume.

### Parameter setting

As to ConvLSTM model, Adam optimization algorithm was adopted to optimize its inherent parameters, and the number of hidden layers and hidden layer nodes were determined by trial and error method, as shown in Table 1. Additionally, the B-spline order *n* and bandwidth threshold *ξ* of TVF-EMD algorithm were set to 26 and 0.25, respectively, considering its decomposition efficiency.

**Table 1:** Parameters and hyperparameters of the proposed model.

Model	Parameter	Value
ConvLSTM	Input shape	(2, 1, 3, 2)
	Filters	{12, 12}
	Kernel size	{(2, 2), (2, 2)}
	Activation function	{‘relu’, ‘relu’}
	Loss function	Mean square error (MSE)
	Optimization algorithm	Adam
	Epoch	1,000
ARIMA	(p, d, q)	(9, 1, 2)

### Evaluation criteria

To evaluate the prediction performance of the proposed ensemble forecasting framework and the comparison models, multiple evaluation metrics are applied in this study. In particular, the mean absolute error (MAE), root mean square error (RMSE) and mean absolute percentage error (MAPE) can effectively reflect the prediction accuracy and fitting effect. The Willmott’s index of agreement (WIA) can reflect the generalization performance of the forecasting model. Their mathematical expressions are presented as follows:


(21) $$MAE=\overset{n}{\underset{i=1}{\sum }}|Y_{i}-{\hat{Y}}_{i}|/n$$

(22) $$\mathrm{RMSE}=\sqrt{\overset{n}{\underset{i=1}{\sum }}{\left(Y_{i}-{\hat{Y}}_{i}\right)}^{2}/n}$$

(23) $$\mathrm{MAPE}=\overset{n}{\underset{i=1}{\sum }}|\left(Y_{i}-{\hat{Y}}_{i}\right)/Y_{i}|/n\times 100\%$$

(24) $$WIA=1-\overset{n}{\underset{i=1}{\sum }}{\left(Y_{i}-{\hat{Y}}_{i}\right)}^{2}/\overset{n}{\underset{i=1}{\sum }}{\left(\left|Y_{i}-\bar{Y}|+|{\hat{Y}}_{i}-\bar{Y}\right|\right)}^{2}$$

where $Y_{i}$ and ${\hat{Y}}_{i}$ are the actual value and forecasting value at time i respectively, n is the number of testing samples, and $\bar{Y}$ represents the average value of the real data.

Furthermore, the Diebold-Mariano (DM) test (Diebold and Mariano, 1995) is introduced to evaluate the significant difference in prediction performance between the proposed framework and the comparison models from a statistical perspective, and the loss function adopts the mean square error (MSE). The null hypothesis is that the MSE value of the test model is greater than or equal to that of the comparison model, that is, the prediction performance of the test model is inferior to that of the comparison model. Assuming that model 1 and model 2 are the two models whose prediction effects need to be compared, the DM statistics can be defined by the following formula:


(25) $$DM=\bar{d}\sqrt{{\left(\overset{n}{\underset{i=1}{\sum }}\left(d_{i}-\bar{d}\right)\right)}^{2}/\left(n-1\right)}$$

where $\bar{d}=\frac{1}{n}\overset{n}{\underset{i=1}{\sum }}d_{i}$,$d_{i}={\left(y_{i}-{\hat{y}}_{1,i}\right)}^{2}-{\left(y_{i}-{\hat{y}}_{2,i}\right)}^{2}$; ${\hat{y}}_{1,i}$ and ${\hat{y}}_{2,i}$ represent the forecasting values of model 1 and model 2 at time i respectively; $y_{i}$ is the real value of the original series at time *i*; n is the number of testing samples. Under the null hypothesis, the DM test value obeys the standard normal distribution.

## Discussion

### Experimental result analysis

To comprehensively and fully evaluate the prediction accuracy and effectiveness of the proposed model (i.e., model ① SED-DDP-TVFEMD-ConvLSTM), some comparative models were designed for experiments from the following aspects:

1. In order to verify the effectiveness of the strategy of introducing search engine data, under the framework of non-decomposition, we compared the model with the preprocessed SED as explanatory variable (i.e., model ③) and the single ConvLSTM model without explanatory variables (i.e., model ⑥).
2. To verify the effectiveness of the dual data preprocessing strategy for search engine data, we established a new comparison model without decomposition (i.e., model ⑤). Specifically, five SEDs without DDP were randomly selected from the original SEDs and synthesized into an integrated SED, which was used as the explanatory variable of ConvLSTM model. Then, the feasibility of DDP strategy was further analyzed by comparing model ③ with model ⑤.
3. To demonstrate the role of the spatial–temporal artificial intelligence model in the proposed forecasting framework, two groups of comparative experiments were designed in this paper. First, in the case of introducing decomposition-ensemble strategy, the model with ConvLSTM as the main prediction method (i.e., model ①) was compared with the model with LSTM as the main prediction method (i.e., model ②). Secondly, in the case of without introducing decomposition-ensemble strategy, the model with ConvLSTM as the main prediction method (i.e., model ③) was compared with the model with LSTM as the main prediction method (i.e., model ④).
4. To verify the feasibility of the decomposition-ensemble strategy, two groups of comparative experiments were designed. First, in the case of taking ConvLSTM as the prediction method, the ensemble forecasting framework based on TVF-EMD algorithm (i.e., model ①) was compared with the non-ensemble forecasting framework (i.e., model ③). Secondly, in the case of taking LSTM as the prediction method, the ensemble forecasting framework based on TVF-EMD algorithm (i.e., model ②) was compared with the non-ensemble forecasting framework (i.e., model ④).
5. In order to prove the comprehensive advantages of the forecasting framework considering search engine data, spatial effect and decomposition-ensemble strategy, the proposed framework (i.e., model ①) was compared with the classical single model (i.e., model ⑦ and model ⑧).

The error evaluation results of the proposed model and the comparison models in two cases are shown in Table 2, and based on the analysis of Table 2, the following conclusions can be drawn:

1. Comparing the prediction results of model ③ and model ⑥, it can be observed that the MAE value, RMSE value and MAPE value of model ③ in Case 1 decrease by 15.79%, 13.03%, and 14.98% respectively, and the WIA value increases by 1.06%; in case 2, the MAE value, RMSE value and MAPE value of model ③ decrease by 15.74%, 9.81%, and 14.73% respectively, and the WIA value increases by 2.19%. The comparison results illustrate that the search engine data can be introduced as a more flexible and fuller data source to make up for the singleness of historical statistical data, and effectively improve the prediction accuracy of the model.
2. Without decomposition, the model with DDP strategy for SED (i.e., model ③) is obviously superior to the model without DDP strategy for SED (i.e., model ⑤). For example, compared with model ⑤, the decrease rates of MAE value, RMSE value and MAPE value of model ③ are 23.62%, 15.54%, and 24.54%, respectively, in Case 1, and 23.30%, 23.99%, and 23.23%, respectively, in Case 2. It is proved that the proposed dual data preprocessing strategy has a great cleaning effect, which can correctly identify the outliers and noise information in the search engine big data, and screen the SEDs that have the ability to explain and predict the demand of air travel, so as to promote the prediction model to obtain better results
3. Based on the introduction of SED and decomposition, the performance of spatial–temporal ConvLSTM model (i.e., model ①) is more outstanding than that of non-spatial LSTM model (i.e., model ②). For example, the RMSE value of model ① is 37.74% lower than that of model ② in Case 1, and decreases by 20.81% in Case 2. Similarly, based on the introduction of SED without decomposition, the performance of spatial–temporal ConvLSTM model (i.e., model ③) is still better than that of non-spatial LSTM model (i.e., model ④). For example, the MAPE value of model ③ is reduced by 28.36% compared with model ④ in case 1, and decreases by 5.77% in Case 2. The comparison results show that ConvLSTM model can better grasp the spatial information in the input data to improve the performance of the model.
4. Based on the ConvLSTM model, the performance of the model with decomposition-ensemble strategy (i.e., model ①) is significantly better than that without this strategy (i.e., model ③). For example, compared with model ③, the MAE value of model ① is reduced by 20.78% in Case 1 and 40.95% in Case 2. Similarly, based on the LSTM model, the performance of the model with decomposition-ensemble strategy (i.e., model ②) is still better than that without this strategy (i.e., model ④). For example, compared with model ④, the WIA value of model ② increases by 0.25% in Case 1 and 3.65% in Case 2. The comparison results show that the decomposition-ensemble strategy can avoid the adverse effects of the market environment to effectively improve the prediction precision.
5. Comparing the proposed framework (i.e., model ①) with the single SVR (i.e., model ⑦) and ARIMA (i.e., model ⑧), it can be found that the performance of the proposed framework is significantly better than that of the single models in both Cases. For example, in Case 1, the MAE value, RMSE value and MAPE value of model ① decrease by 74.61%, 70.80%, and 73.74%, respectively, compared with model ⑦, and decrease by 83.80%, 82.55%, and 83.87% compared with model ⑧. The comparison results fully prove the comprehensive advantages of the proposed framework, which not only plays the role of search engine data and spatial effect, but also combines the data analysis ability of different models.

**Table 2:** Prediction results of all the models.

Case	Model	MAE	RMSE	MAPE (%)	WIA
Case 1	① SED-DDP-TVFEMD-ConvLSTM	**1.5068**	**2.0332**	**0.7209**	**0.9866**
	② SED-DDP-TVFEMD-LSTM	2.1629	3.2654	1.0444	0.9657
	③ SED-DDP-ConvLSTM	1.9021	2.5782	0.8985	0.9808
	④ SED-DDP-LSTM	2.6234	3.3312	1.2542	0.9633
	⑤ SED-ConvLSTM	2.4902	3.0527	1.1908	0.9694
	⑥ ConvLSTM	2.2587	2.9644	1.0568	0.9705
	⑦ SVR	5.9349	6.9635	2.7454	0.8268
	⑧ ARIMA	9.3035	11.6494	4.4684	0.2080
Case 2	① SED-DDP-TVFEMD-ConvLSTM	**1.8607**	**2.4502**	**0.8872**	**0.9796**
	② SED-DDP-TVFEMD-LSTM	2.3514	3.0941	1.1289	0.9667
	③ SED-DDP-ConvLSTM	3.1509	4.0562	1.5147	0.9495
	④ SED-DDP-LSTM	3.4039	4.2216	1.6074	0.9327
	⑤ SED-ConvLSTM	4.1079	5.3361	1.9730	0.9097
	⑥ ConvLSTM	3.7394	4.4973	1.7763	0.9292
	⑦ SVR	9.1966	10.3618	4.3166	0.6704
	⑧ ARIMA	9.1321	10.9896	4.3730	0.3980

The best results are bold.

In this paper, DM test was employed to further evaluate the superiority of the proposed forecasting framework from a statistical perspective by revealing the performance gaps between proposed model and the comparison models, respectively. The calculation results under Case 1 and Case 2, respectively, are presented in Table 3.

**Table 3:** DM test results of the proposed model and the comparison models.

Comparison model	Case 1		Case 2	
	DM-statistic	Prob.	DM-statistic	Prob.
② SED-DDP-TVFEMD-LSTM	−1.9109*	0.0660	−2.4662**	0.0163
③ SED-DDP-ConvLSTM	−1.5064	0.1428	−4.6252***	0.0000
④ SED-DDP-LSTM	−3.5643***	0.0013	−5.2239***	0.0000
⑤ SED-ConvLSTM	−3.9033***	0.0005	−5.0077***	0.0000
⑥ ConvLSTM	−2.8186***	0.0086	−6.1822***	0.0000
⑦ SVR	−5.5977***	0.0000	−11.6820***	0.0000
⑧ ARIMA	−6.2229***	0.0000	−9.2150***	0.0000

*** is the 0.01 significance level, ** is the 0.05 significance level, * is the 0.1 significance level, and the MSE is taken as the loss function.

As illustrated in Table 3, the DM statistics of all comparison models are <0. Moreover, except for models ② and model ③, the proposed forecasting framework is significantly better than other comparative models at a significant level of 0.01 in Case 1; and have the same performance in Case 2 except for model ②. Conclusively, the proposed forecasting framework has excellently statistical validity.

### Deep insights

The previous analysis of the experimental results proved that the application of Internet big data and spatial information has a prominent effect on improving the accuracy of demand forecasting. Based on this research achievements, some deep insights on how the relevant bodies can benefit from it have produced.

For the government, they can organize the establishment of a tourism big data information sharing platform to achieve the linkage of public health, crisis early warning, crisis decision-making and other public management systems. Based on the specific predicted demand, a comprehensive and specific emergency response plan need be prepared to improve the public risk prevention and control capability. In addition, government is advised to strengthen the positive publicity of the effect of the safe travel policy to reduce the public’s concern about the risk of long-distance travel. Meanwhile, they can strengthen the scientific literacy education related to the crisis by Internet, further enhance the citizens’ scientific cognition of the effectiveness of crisis prevention and control, so as to alleviate their negative emotions. Additionally, based on the capacity of each scenic spot and the predicted results, government should not only arrange corresponding health facilities, but also complete the advance scheduling of resources (e.g., public transport, consulting services and medical services, etc.), to provide tourists with a sense of security for traveling. And preferential policies also need to be introduced to stimulate the tourism industry and drive economic development periodically.

For tourism companies, they better to fully realize information sharing among tourism enterprises, which is conducive to reducing the cost of enterprise information collection, so as to make accurate response to sudden crises. Moreover, according to the characteristics of varying demands, tourism service quality and passenger satisfaction can be improved by optimize resource allocation, strengthen human resource training and reshape service processes. And they can further analyze and respond new travel needs of the public, such as safety, health and convenience, through timely adjustment and optimization of the enterprise’s operational objectives and work ideas based on the scientific prediction of travel demand. Meanwhile, it is critical to pay attention to the destinations with high travel intention of passengers, deepen the exploration of new tourism resources, or effectively integrate existing tourism resources combined with big data mining technology. This method caters to people’s inherent pursuit of novelty, which is beneficial to the transformation of public positive emotions.

## Conclusion

Nowadays, the social instability factors emerge in endlessly, has led to the global social psychological problems. In order to give full play to the role of long-distance travel in struggle against public anxiety and negative, starting from the search behavior related to travel behavior, this paper put forward an ensemble forecasting framework for air travel demand based on big data mining technology and artificial intelligence algorithm, and applies it to predict the air travel demand of Shanghai Pudong International Airport. The experimental results show that the proposed framework, adding search engine data with effective data preprocessing, employing the superior ConvLSTM model to mine the spatial–temporal information in the input data, and combining the advantages of different models, can alleviate the inherent content complexity of big data and obtain more satisfactory prediction results than the comparison models. The high performance of the proposed forecasting framework means that individuals and relevant departments can grasp the travel demand of passengers in advance, and make reasonable short-term planning and risk control for airports, scenic spots, hotels and so on, which greatly improves the sense of safety of passengers. On the basis of the achievements of this study, several suggestions about the formulation of policies and measures for the government and tourism companies in the unstable era are put forward for reference, which thoroughly provide a fuel for promoting travel safety, tourism revitalization and public anxiety alleviation.

In addition, although the proposed model has achieved good prediction results, there are still gaps to be filled. For example, this paper only conducted empirical research on the relevant actual data of Shanghai Pudong Airport, and more airport samples should be considered in the subsequent research. Moreover, the framework included only search engine data. In future researches, more types of big data can be incorporated into the model, such as news topics and comments on social media. Finally, this paper employed the trial and error method to determine the parameters of ConvLSTM model, which had a negative impact on the stability and convenience of the model. In the subsequent research, advanced optimization algorithms can be used to achieve automatic optimization of parameters.

## Data availability statement

Publicly available datasets were analyzed in this study. This data can be found at: https://github.com/QingZ96/Air-Passenger-Demand-Forecasting.

## Author contributions

XL devised the study and collected the data. CH analyzed the data and wrote the manuscript. MY and WZ helped with writing and editing the manuscript. All authors contributed to the article and approved the submitted version.

## Funding

This research was funded by the National Natural Science Foundation of China (nos. 71701122 and 11801352).

## Conflict of interest

The authors declare that the research was conducted in the absence of any commercial or financial relationships that could be construed as a potential conflict of interest.

## Publisher’s note

All claims expressed in this article are solely those of the authors and do not necessarily represent those of their affiliated organizations, or those of the publisher, the editors and the reviewers. Any product that may be evaluated in this article, or claim that may be made by its manufacturer, is not guaranteed or endorsed by the publisher.

## Footnotes

^1^ http://www.wind.com.cn/

^2^ https://index.baidu.com/
